# Exercise Condition Sensing in Smart Leg Extension Machine

**DOI:** 10.3390/s22176336

**Published:** 2022-08-23

**Authors:** Yaojung Shiao, Thang Hoang

**Affiliations:** 1 Department of Vehicle Engineering, National Taipei University of Technology, Taipei City 106344, Taiwan; 2 Center of Electrical Engineering, Duy Tan University, Danang 550000, Vietnam

**Keywords:** fitness exercise, rehabilitation exercise, leg extension, muscle fatigue, electromyography

## Abstract

Skeletal muscles require fitness and rehsabilitation exercises to develop. This paper presents a method to observe and evaluate the conditions of muscle extension. Based on theories about the muscles and factors that affect them during leg contraction, an electromyography (EMG) sensor was used to capture EMG signals. The signals were applied by signal processing with the wavelet packet entropy method. Not only did the experiment follow fitness rules to obtain correct EMG signal of leg extension, but the combination of inertial measurement unit (IMU) sensor also verified the muscle state to distinguish the muscle between non-fatigue and fatigue. The results show the EMG changing in the non-fatigue, fatigue, and calf muscle conditions. Additionally, we created algorithms that can successfully sense a user’s muscle conditions during exercise in a leg extension machine, and an evaluation of condition sensing was also conducted. This study provides proof of concept that EMG signals for the sensing of muscle fatigue. Therefore, muscle conditions can be further monitored in exercise or rehabilitation exercise. With these results and experiences, the sensing methods can be extended to other smart exercise machines in the future.

## 1. Introduction

Exercise can improve the quality of life because it helps people reduce the risk of some diseases and lose body weight. Several fitness and rehabilitation exercises are suitable for personal health. Recently, some active or semi-active exercise machines have been developed to provide adjustable loads and smart functions to users. These machines significantly minimize the disadvantages of conventional heavy machines for fitness or rehabilitation. Weightlifters largely pursue the development of lean human mass. For example, a leg extension machine is used to train the quadriceps by flexion and extension of the knee. Various devices have many useful applications in rehabilitation or training exercises to develop body tissues. We have designed a new smart leg extension to improve the conventional machines [1]. The resistance training program that is exercise-induced develops the muscles [2,3,4,5]. Muscular strength is a primary target for athletes in strength and power sports, such as rugby, football, and fitness exercise. Exercise induces muscle hypertrophy to include three factors to develop the muscle, namely, mechanical tension, muscle damage, and metabolic factors [6]. Based on the characteristics of muscle hypertrophy, the force created by the leg extension creates a motion pathway of the leg and a natural feeling. We utilize these to make the machine smart. The various effects relate to the smart material technology of magneto-rheology (MR) to be applied in the resistance device [7,8,9]. Moreover, the signal from the human helps to observe the conditions of the training exercise.

By tracking real-time user behavior with this machine, several phenomena were observed in this study. We focused on the development of the sensing technology of user operating conditions on a smart leg extension machine. There are many kinds of signals that can sense personal health conditions during an exercise. For example, electromyography (EMG), a saturation of oxygen (StO2), and electroencephalogram (EEG) signals of people are useful in detecting muscle conditions. These signals aid the understanding of human factors in evaluating exercise quality. This exercise can be more efficient or can prevent accidental injury. Oxygen is metabolically vital to biomechanically foster oxidative phosphorylation to form ATP, and lack of oxygen in metabolic pathways is converted to lactate that has been implicated in muscle soreness [10,11,12,13,14]. Saturation of oxygen (StO2) is the estimated amount of oxygen in the blood by emitting and then absorbing a light wave passing through blood capillaries [15]. The EEG is the signal from the brain that has been well studied to find the feature to diagnose some diseases. It is also applicable in detecting muscle fatigue, Ali A. Abdul-Latif performed analyses on EEG by evaluating the root mean square of EEG bands after fatigue [16]. EMG has been developed and used to evaluate the effect of condition training muscle and muscle fatigue by algorithm signal processing with data collected from a part of the body [17,18,19,20]. Triwiyanto and Wahyunggoro performed extraction using the Wilson amplitude (WAMP) and mean frequency to recognize the fatigued muscle [21]. Likewise, Abdulhamit and Kema investigated this phenomenon in time-frequency combination with independent component analysis (ICA), and they used the wavelet transforms to determine fatigue [22]. Further, Shair et al. presented methods to study muscle fatigue through EMGs processing in time and frequency domains [23]. Consequently, EMGs can be used to analyze the state of muscles. For example, the information on muscle fatigue can be seen in the frequency of EMGs. However, there are not enough studies on the index conditions of the muscle and the factors that affect the muscle during practice exercises with smart machines.

In this study, we investigated the monitoring muscle fatigue condition during exercising by observing EMG signals. During the exercise using a smart machine, the resistance torque effect on the leg affects the state of the muscle. The purpose of this study was to develop a new index to track the exercise conditions of people using EMG. We used EMG signals because they are directly created by muscle contraction. Users are detected in real-time with muscle alternation. If the training strength is too high, the user will risk receiving muscle injury, which can be fatal. Moreover, real-time training results are vital for instructors or therapists to evaluate users’ conditions. Therefore, user condition sensing is very critical for the applications of smart exercise machines. For the first time, however, our aim was to sense muscle fatigue effectively by simple sensors. Therefore, a new method was adopted by checking the wavelet signals of EMG signals. 

## 2. Materials and Methods

### 2.1. Muscle Model and Condition Sensing

**Figure 1:**
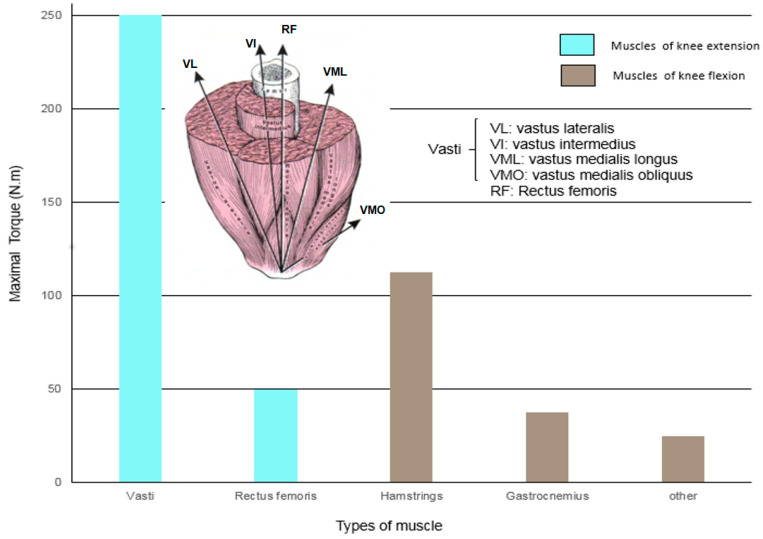
The maximum torque at muscles across the knee.

Three types of leg muscle contractions are required to perform various knee functions. First, isometric contraction protects and stabilizes the knee. Second, eccentric contraction provides shock absorption to the knee. Third, concentric contraction accelerates the femur into the knee and creates the action to raise the body’s center of mass. The quadriceps are a large and powerful extensor muscle which contains many types of muscle, such as the rectus femoris (RF), vastus lateral (VL), vastus medialis (VM), and vastus intermedius (VI). From the data in the research of Melissa and Felix [24], we made a bar chart in Figure 1 that shows the relationship between the maximal torque and endurance of each muscle when humans perform an action in the leg for extension or flexion. The vasti and RF muscle is the main muscle that undergoes the force of leg extension.

**Figure 2:**
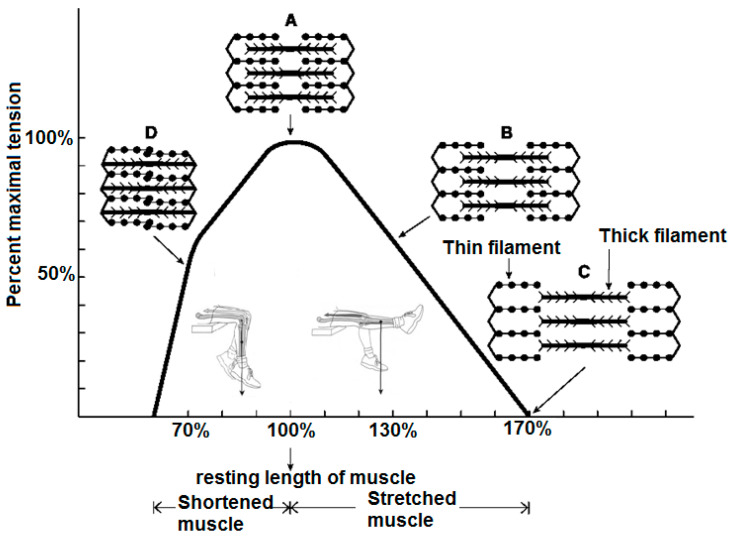
The relationship between the tension and length of a muscle.

In Figure 2, A–D are the states of tension of muscle fiber length. The maximum tension of muscle fiber length peaks at point A because the range at this point can make a maximal number of cross-bridges, and actin molecules are accessible to one another for a cycle of blending and blinding [25].

The properties of muscles provide methods to aid the design of good training functions by controlling the resistance of the leg extension machine. The new technology of MR fluid offers a good performance to make the alternative curve following the angle of leg in which the torque is created by a machine similar to the curve of the feature muscle above. The maximum value of torque depends on the condition exercise to set up the mode for the machine. In this study, we want to find the index for a muscle to control the curve with a maximum torque of 85 Nm, which will reduce to adapt the state of the leg muscle by the index indicating the condition.

**Figure 3:**
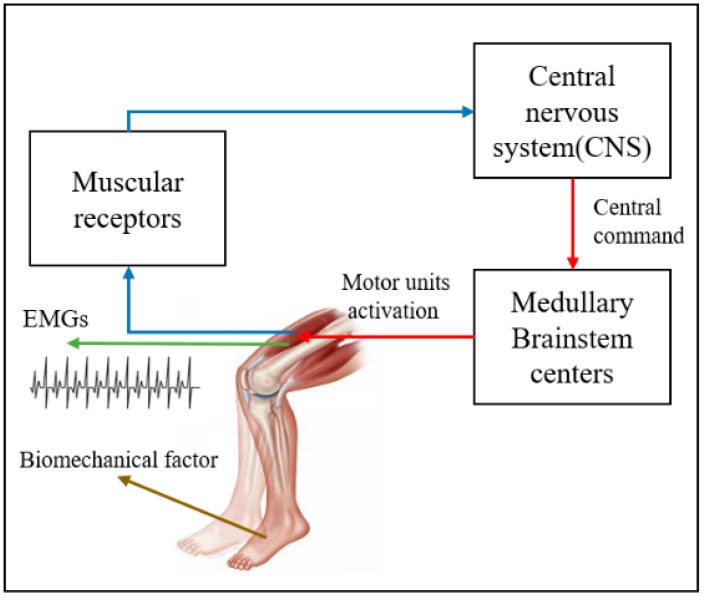
EMGs associated with the generation of muscle force.

The EMG signals are the results of the transmission of electrical motion at the neuromuscular junction where a motor neuron is activated. Nerve stimulation refers to the ability of a muscle to generate the force that stimulates muscle fibers and creates muscle strength. Figure 3 illustrates the EMGs associated with the generation of muscle force.

The relationship between EMG and muscle condition has been investigated in numerous studies in which authors discussed various indices of the exhaustion of the musculoskeletal system [26]. In the general motion of the human leg, the frequency ranges from 0 to 500 Hz [27]. The RF muscle has a range of frequency from 50 to 250 Hz, and it is on people walking and standing. Fatigue can be determined by analyzing the EMG signal, and we define muscle fatigue as a decrease in the force-generating capacity of a muscle. This kind of change in mechanical performance capacity results in EMG changes. The effect of fatigue is observable in the recorded EMG signal as a change in the values of selected EMG parameters. These parameters can be achieved by processing the EMG signal in the time and frequency domains. To survey the EMGs, there are many methods to transform the muscle signals from the time domain to the frequency domain or time-frequency domain. Table 1 shows the methods of processing these signals. In our research, the wavelet packets are applied to calculate entropy. This is the advanced method to analyze the EMGs, which compares to the traditional method referred to as Fast Fourier Transform (FFT) in the previous research. Comparison by using Fourier and wavelet: Fourier only sees on the frequency domain, but wavelets can see both time and frequency. Wavelet waves have shapes similar to EMGs, so it is easy to describe the properties of EMGs.

### 2.2. Evaluating the Real-Time Index Condition Extension

#### 2.2.1. Participants

This study involved 5 healthy males who performed exercises with basic actions following the rule for fitness. The average age was 24 ± 2 years, and their health was normal. The information of them is showed in Table 2. We collected the EMG signals from them when they performed leg extension on the machine. They all followed the rule in leg extension fitness to ensure that conditions were the same for everybody.

Step 1: Put the leg under the crossbar at the front of the machine;

Step 2: Maintain breathing, stretch legs, move legs from low to high;

Step 3: Return to the low space from the height to low. Follow step 2 and 3.5~20 times and rest for 3~10 min;

Step 4: End the exercise.

#### 2.2.2. Wavelet Packet Transform and Wavelet Entropy

In this study, we used the analytic wavelet transform and the entropy of wavelet packets to analyze the EMG signals and present the features. Generally, there are two classes of wavelet transform, namely, continuous wavelet transform and discrete wavelet transform (DWT). The Daubechies wavelet analysis is a kind of discrete wavelet transform, which is called father wavelet, to determine the right scaling, scale-independent method, and can be seen as a diagram time-frequency domain.

(1) $$\phi \left(x\right)=\sqrt{2}\sum_{k=0}^{N-1}c_{k}\phi (2x-k)$$

In this formula, ϕ(x) known as the scaling function (or father wavelet) and is the creating block for the wavelet basis. The equation is the dilation equation, which defines the scaling function and wavelets. Wavelet families have many kinds of wavelet functions, for example: Haar, Deaubechies, Biorthogonal. Daubechies has a shape similar to the signal of EMG more than another wavelet function, so it is easy to describe the shape of EMG by scales with high accuracy. Moreover, the Daubechies wavelet uses overlapping windows, thus the results reflect all changes.

The discrete wavelet packet transform (DWPT) is an extension of DWT. It is created by the generalization of the link between wavelet transforms and multiresolution analysis. The hypothesis is that the *h_k_* and *g_k_* quadrature mirror filters corresponding to the mother wavelet and the scaling function are two sequences *l*^2^ (Z). The wavelet packet is expressed thus:

(2) $$\Psi_{2n}\left(t\right)=\sqrt{2}\underset{k\in Z}{\sum }h_{k}\Psi_{n}\left(2t-k\right)$$

(3) $$\Psi_{2n-1}\left(t\right)=\sqrt{2}\sum_{k\in Z}g_{k}\Psi_{n}\left(2t-k\right)$$

**Figure 4:**
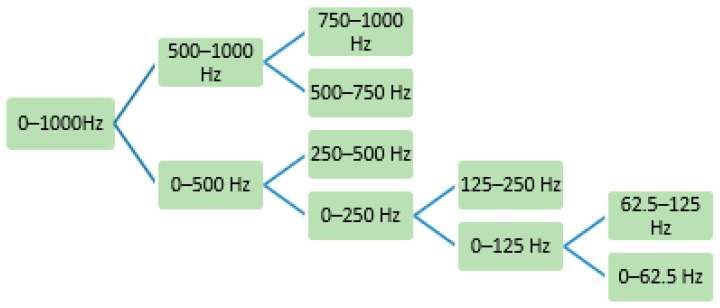
Flowchart of 4 levels of DWPT.

For the DWPT, Figure 4 shows the packet in the tree at level 1, from 0 to 500 Hz as the low-frequency part, and the other as the high-frequency part. In the DWPT, the detail and the coefficients at each level are designed by passing the range of frequency packet similar to the binary tree.

Wavelet entropy is a presentation of uncertainty in the entire signal duration, and it can measure the imbalance of the system. This implies that we will observe the situation of the EMG during training on the leg extension machine. The Shannon entropy produces a useful criterion for analyzing the system disorder. Following the theory of entropy given by Shannon, the wavelet packet entropy is expressed by the continuous equation:

(4) $$H\left(x\right)=-\int_{-\infty }^{\infty }p\left(x\right)\log\left[p\left(x\right)\right]dx$$

#### 2.2.3. Data Processing

**Figure 5:**
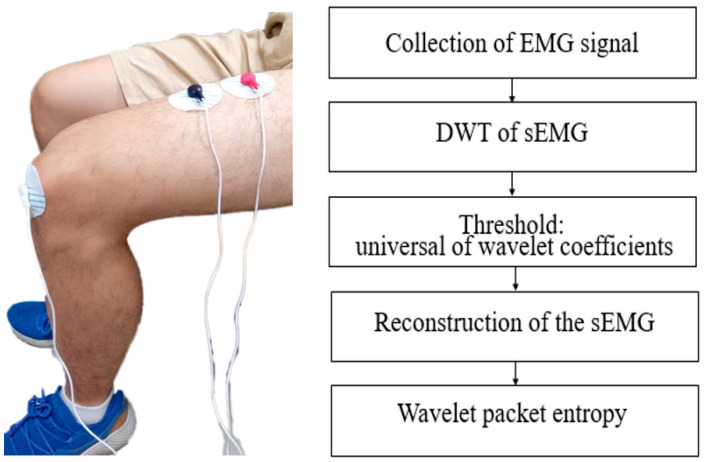
Processing the EMG signal to entropy.

We used the EMG sensor of Biometrics Ltd. (SX230-1000; Newport, UK). The specification of the sensor has one parameter focusing on the integrated filter inside. We put the device of EMG on the thigh of the leg because of collecting EMGs from the leg muscle. The EMG signals were recorded by using NI 6221 data acquisition. The signal of EMG is in the range from 0 to 500 HZ as mentioned above, thus we chose the sampling frequency of 1000 Hz because the Nyquist sampling theorem for sampling frequency requires it to be at least twice the highest frequency contained in the signal. It is known that the DWT of EMG signals is estimated by a series of filters with the wavelet transform coefficients. The signals, including the white noise, were collected by DAQ NI 6221. The white noise energy is distributed in the whole wavelet domain (time and frequency domain). To reduce the white noise, we used the wavelet de-noising algorithm. The algorithm supposes a finite length signal that superposes a Gaussian white noise, and it is expressed as: sEMG(t) = semg(t) + n(t). The formula shows the original EMG signal (semg(t)) and the white noise n(t). We recovered the original signal semg(t) from sEMG(t) and contained the noise pollution n(t) by processing step-by-step, as shown in Figure 5. It has three basic steps: (1) By using the father wavelet to make wavelet decomposition, obtain corresponding wavelet coefficients, (2) select the threshold for coefficients, (3) make the inverse transform to restructure the EMG signal. After the signal was removed from the noise, we analyzed the wavelet packet entropy on the signals. The properties of fatigue muscle include changing the range of frequency from 250 to 60 Hz, thus we selected level 4 at the packet with frequency from 125 to 62.5 Hz. The figure gives us all the processes to make the entropy of sEMG.

#### 2.2.4. Hardware Design

**Figure 6:**
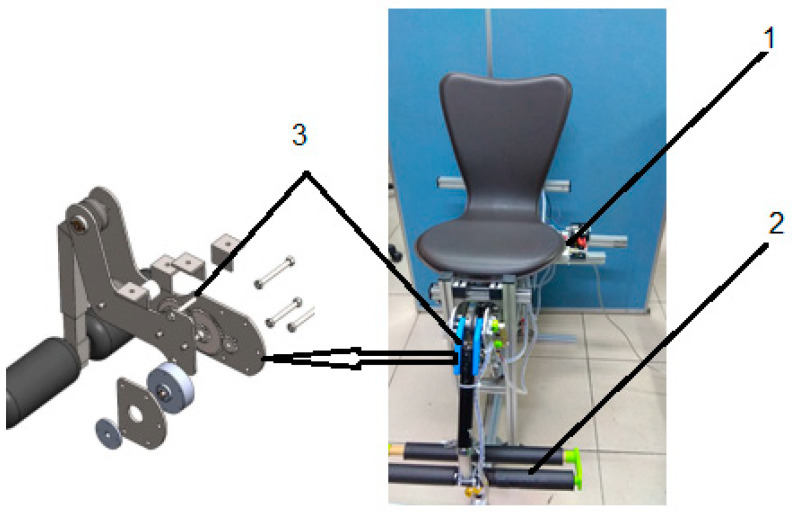
Leg extension machine model (1. The module collects the data from users; 2. force sensor; 3. variable resistance).

We designed the leg extension machine in Figure 6 relative to the model properties of the muscle. To make it more suitable, we propose many curves torque to feasible with the behavior of personal training during the time. The algorithm control will change the torque trajectory in the machine.

**Figure 7:**
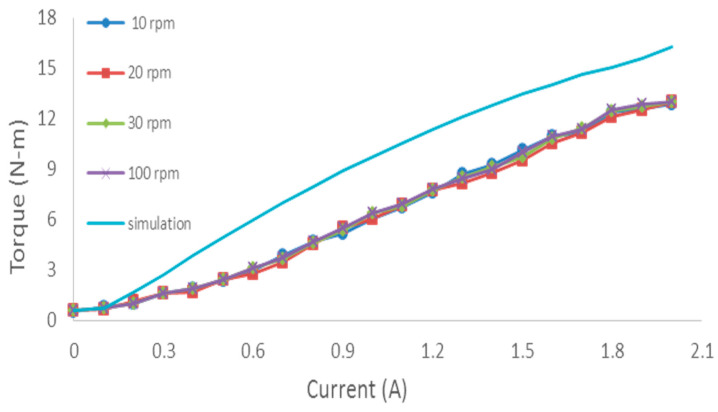
The relationship between resistance torque of the MR brake and current.

The new technology is applied in this device (MR brake) to create resistance. MR fluid filled all the gaps inside the device. The controller provides the current into the coil of MR in which the magnetic field appears around the coil to vary the viscosity of MR fluid. The rheological or smart material under the application of a magnetic field creates yield strength that the MR fluid will utilize to transform the state with different viscosities, but the viscosity with a current is linear. Figure 7 shows the relationship between the torque and current of the device. When a person uses this device, changing the load changes the current applied to the MR brake. Therefore, the change of load is continuous without interruption during exercise, making the training process more efficient. It depends on the condition of the muscle through the muscle fatigue index that changes the load.

#### 2.2.5. Acceleration and Entropy Detection Analysis

In this experiment, we used the accelerometer to estimate the frequency of leg contraction exercise. When the accelerometer was placed on the foot and the foot was up and down, the Z-axis signal had the largest change compared to the signals in the other two axes. The integrated signal from the *Z*-axis of the accelerometer sensor by the leg-lifting force created the value (*Z*-axis acceleration value). When the lift of the participant finished, we obtained the frequency and the value of *Z*-axis by definition of frequency. From the sample time of *Z*-axis data, the frequency equals one divided by the time period. The number of leg contractions was compared with the EMG entropy to find the characteristic signals of muscle fatigue.

**Figure 8:**
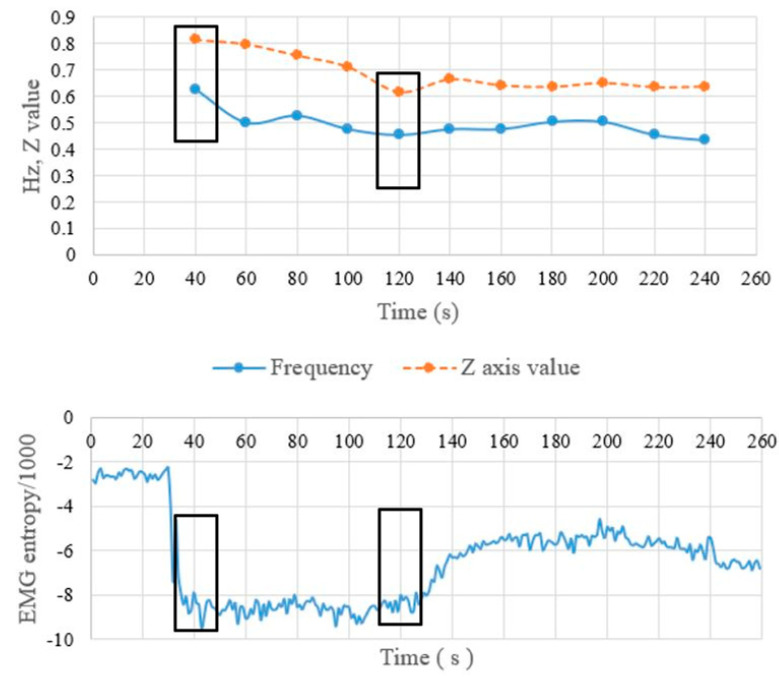
Comparison of *Z*-axis acceleration frequencies and entropy values (**top**). Number of leg lifts and leg lift force (**bottom**) EMG entropy values.

We illustrate an empirical example of the relationship between frequency and EMG entropy. As shown in Figure 8, we used the result of one user to explain the relationship between the value of the *Z*-axis acceleration and EMG entropy value. The first block of the two charts is at time 30 s to 50 s during which the number of legs contractions (frequency) is 0.625 Hz, and the *Z*-axis acceleration value is 0.8135. The second block is at time 130 s in which the number of leg contractions (frequency) is 0.454 Hz, and the *Z*-axis acceleration value is 0.6154. It means that the frequency and *Z*-axis acceleration of a leg doing extension decreases with time. When the frequency moves to the limit point, the muscle becomes fatigued. The contractions are few, and the force for the leg lift is also lowered. That is the symptom of muscle fatigue. Hence, the muscle fatigue of the users can be inferred by recognizing that the EMG entropy value appears when the user develops muscle fatigue.

In this study, the EMG sensor is used to capture EMG signals from the leg muscle. The chemical balance affects the transmission of motor neuron signals due to changes in EMG signals, and it has a negative effect on the neurons of the muscle cells [26,27,28,29]. Muscle fatigue causes the spectrum EMG signals to shift to a low frequency for everyone that has been synthesized and concluded in the study of Merlo et al. [30,31,32,33].

#### 2.2.6. Detection and Analysis of Entropy Values under Different Loads

The smart leg extension system was used under different loads and users to practice leg extension with testing loads of 4.5, 11, and 18 kg. This section detects and analyzes the users’ muscle fatigue through the relationship between EMG entropy and muscle fatigue under different loads. To ensure correctness and safety, we set up each person to run one experiment per day, with each test lasting for 5 min for the safety of the person and to avoid incorrect data due to fatigue (if the person was uncomfortable to complete it due to fatigue, the experiment was terminated early). We analyzed the experimental data and made comparisons.

Further, we used a nonlinear digital filter technique, which is the median filter, to remove the noise of the signal, check the sample input signal, and determine whether it represents the signal. The intermediate value of the sorting is the output. The filter is to make a smooth curve for these signals and to recognize parameters T1, T2, and T3.

**Figure 9:**
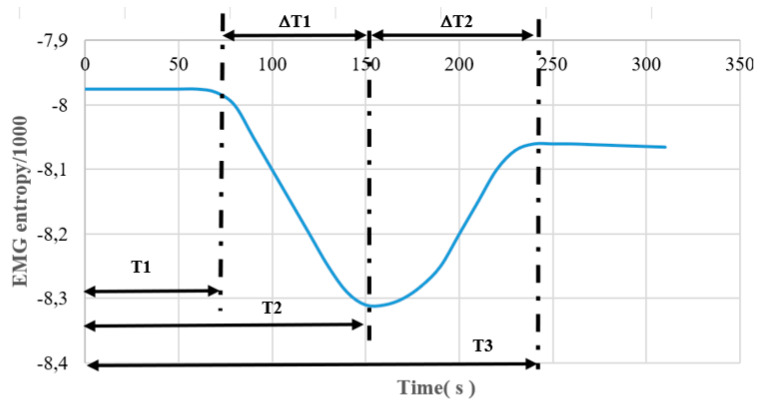
Positions of T1, T2, T3, ∆T1, and ∆T2.

Specifically, we defined the location of T1, T2, T3, ∆T1, and ∆T2. As shown in Figure 9 below, T1 represents the point when the EMG entropy value begins to decrease after the person starts to move. T2 is the point when the EMG entropy value reaches the least valley. T3 is the point after the EMG entropy value starts to rise to the highest point. ∆T1 is the time difference between T2 and T1, and ∆T2 is the time difference between T3 and T2.

**Figure 10:**
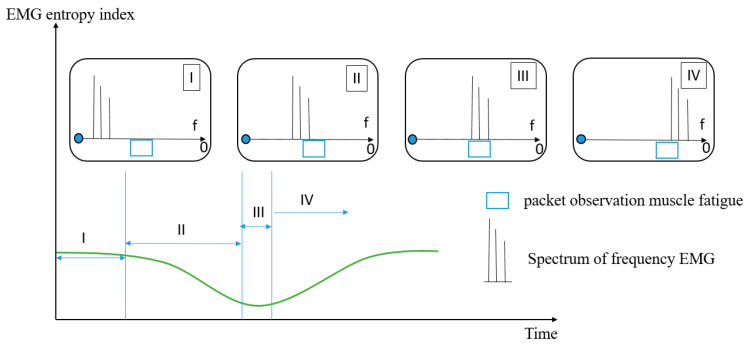
The relationship between spectrum of frequency and EMG entropy index.

In the research, two sensors were used to detect muscle fatigue (IMU and EMG sensor). The mission of IMU is to verify the condition of muscle, when a person has muscle fatigue, the frequency will decrease over time. The EMG entropy from analyzed the EMG sensor is a new index of muscle fatigue. The spectrum of EMG will be decreasing, as mentioned above. Although it will be moving from 250 to 0 Hz during muscle fatigue, we chose the packet from 125 to 62.5 Hz as the fatigue state of the muscle. Figure 10 illustrates the spectral shift of EMG in the frequency domain corresponding to the EMG entropy curve in which I–IV are the cases of location of spectrum. In section I, the peak of spectrum EMG distributes more than the frequency of packet observation muscle fatigue (PKO), so the EMG entropy does not seem to have changed much. In section II, when the spectrum of EMG begins entering the PKO, the EMG entropy will be declined. In sections III and IV, the peak of spectrum EMG gradually left the PKO, so it gradually increases again.

The participants in the experiment were limited to the group of young males because we sought to create a new index of tracking muscle state by using wavelet packet entropy. Although the EMG signals vary by individuals, the properties of spectrum frequency that shift to the low-frequency region are the same, which has been confirmed as mentioned in the introduction about muscle fatigue.

## 3. Results and Discussion

### 3.1. Observation Results

**Figure 11:**
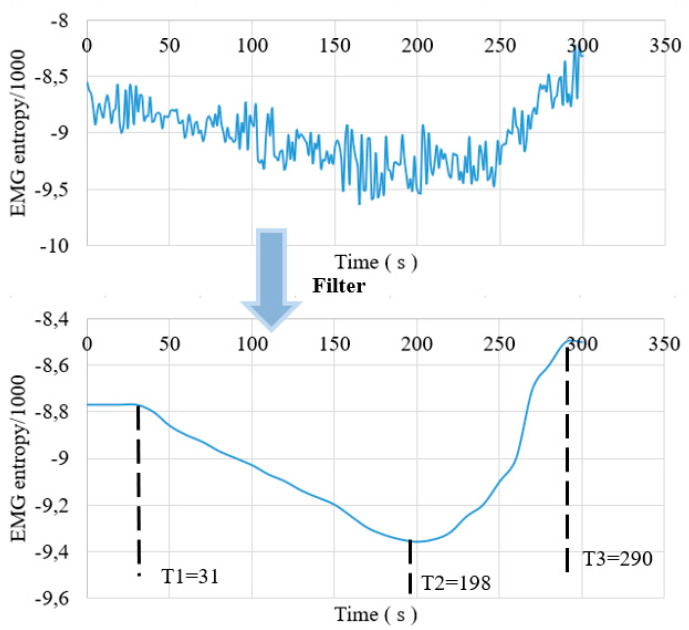
The EMG entropy of experimental data of subject A at 4.5 kg.

The entropy value of the following data has been scaled for readability. We conducted tests on a person for 6 days and with different loads. The test data are shown in Figure 11, which is the original EMG entropy and smooth curve EMG entropy of filtering one person.

**Figure 12:**
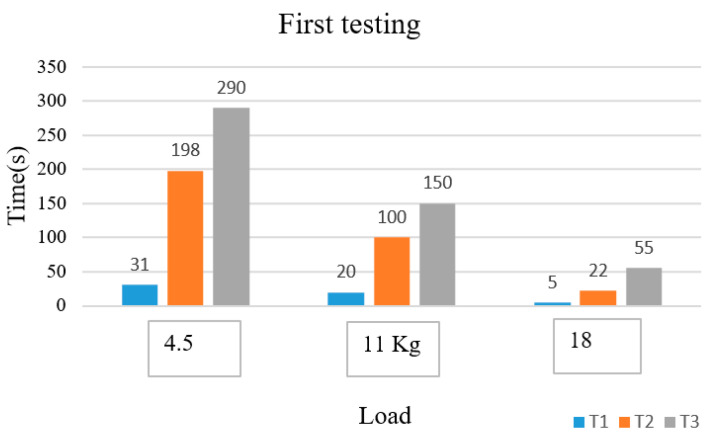
Parameters of time fatigue with different loads in the first testing.

**Figure 13:**
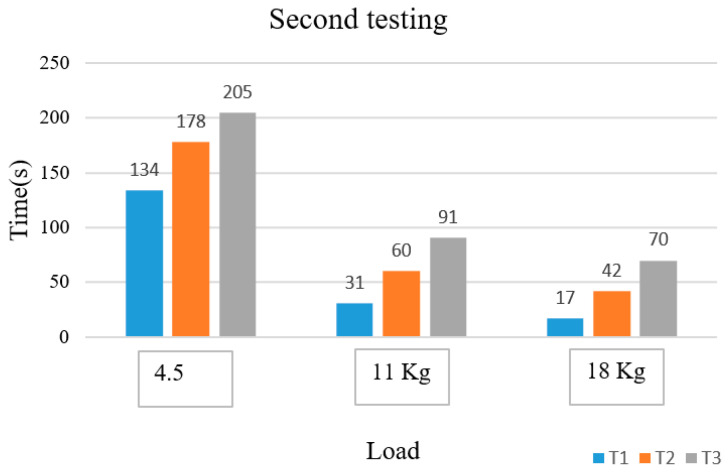
Time fatigue parameters with different loads in the second testing.

From Figure 11, we see the entropy of EMG describing muscle fatigue. The lactic acid starts to accumulate at time T1, and the muscle is fatigued at the time T2. Time T1 is 31 s with entropy of −8.77, and time T2 is 198 s with entropy of −9.36. Time T3 is 290 s with entropy value of −8.5, ∆T1 is 167 s and ∆T2 is 92 s. We conducted experiments with different loads and twice for each person. The results in Figure 12 and Figure 13 below show the behavior character of fatigue through the parameters T1, T2, and T3.

**Figure 14:**
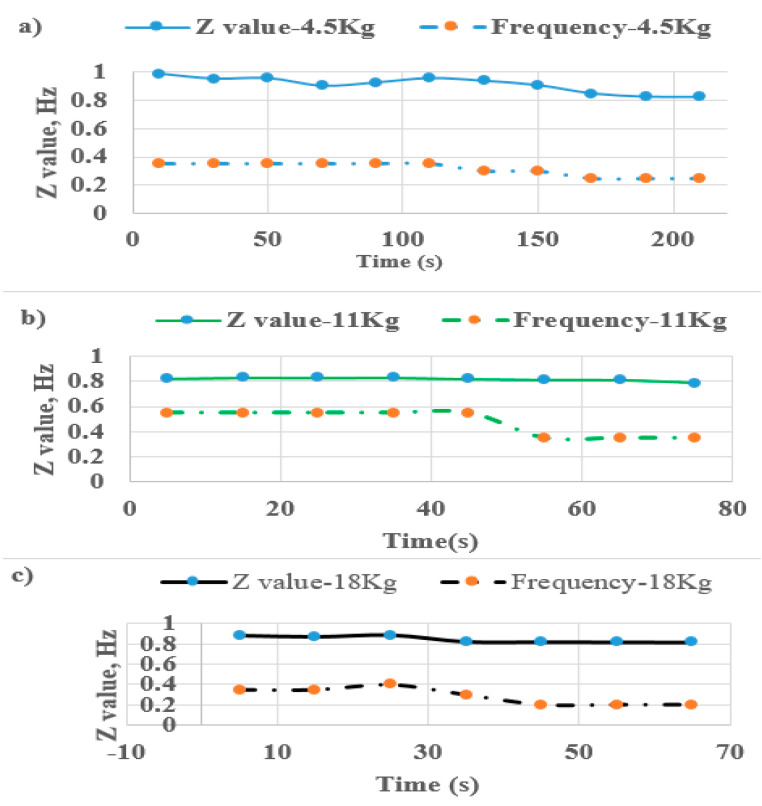
Leg raising frequency and force of subject A under various loads: (**a**) 4.5 kg, (**b**) 11 kg, (**c**) 18 kg.

Figure 14 shows the frequency of exercise motion (f_m_) and Z value of IMU sensor with different loads applied to the leg during training on the leg extension machine. The Z value represents the force or power of the human progressing. The muscle fatigue compared with EMG entropy occurs by observing the frequency reduction.

**Figure 15:**
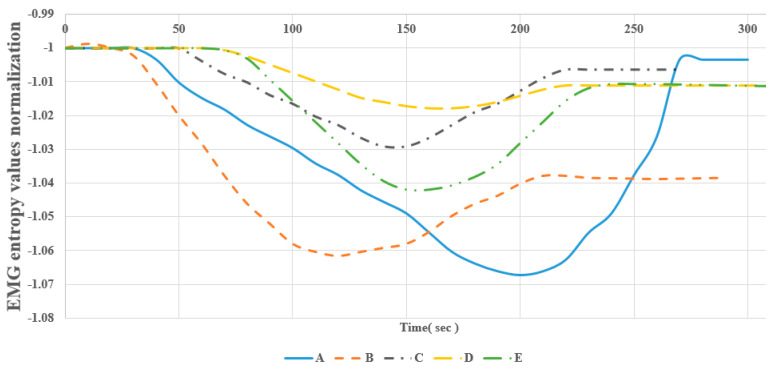
Comparison of entropy values of subjects under 4.5 kg load.

**Figure 16:**
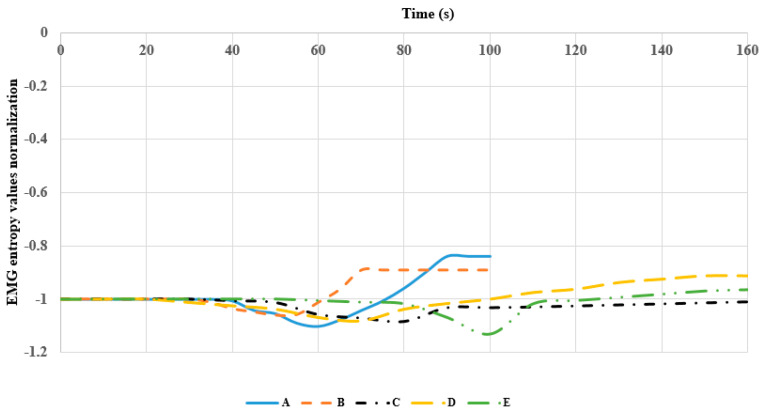
Comparison of entropy values of subjects under 11 kg load.

**Figure 17:**
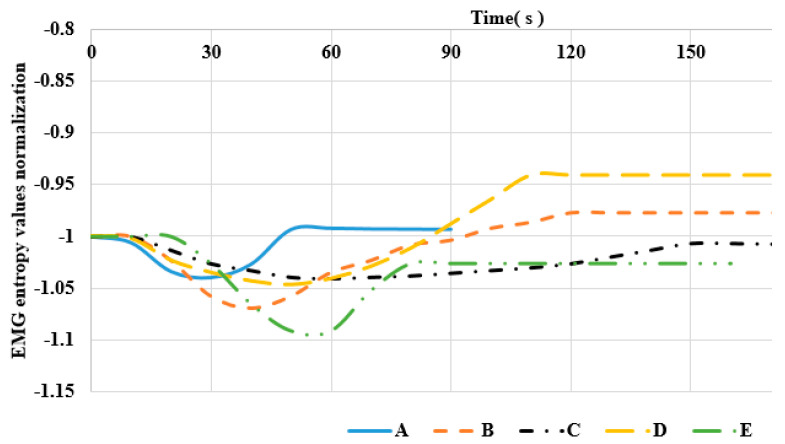
Comparison of entropy values of subjects under 18 kg load.

LabVIEW has the median filter for the signal analysis. We used the median filter to make the smooth curve with time. Figure 15, Figure 16 and Figure 17 show the EMG entropy after passing the median filter, and A-E are the participants in the testing (in Table 2). For obvious comparison of objects, they have been normalized the same EMG entropy of starting value during the period time of exercise. The end points EMG entropy of the members differed because the duration of muscle fatigue of the subjects was different.

Moreover, Figure 15, Figure 16 and Figure 17 show that the EMG entropy of each person changes over time, while the leg extension machine training starts working, following the progression of the loads from 4.5 to 18 kg. The features are represented by time from the bottom to the top and performance condition of the leg muscle leading to muscle fatigue. Each person’s physical fitness was different, thus the time of fatigue was different, and it was not based on the nature of judgment. The trend of the people from non-fatigue to fatigue describes the bottom-to-top curve. The frequency packet to observe is from 62.5 to 125 Hz, and it falls within the range of the frequency of the muscle. The reduction in entropy means that the distribution of EMG frequencies to converge at the packet is selected.

**Figure 18:**
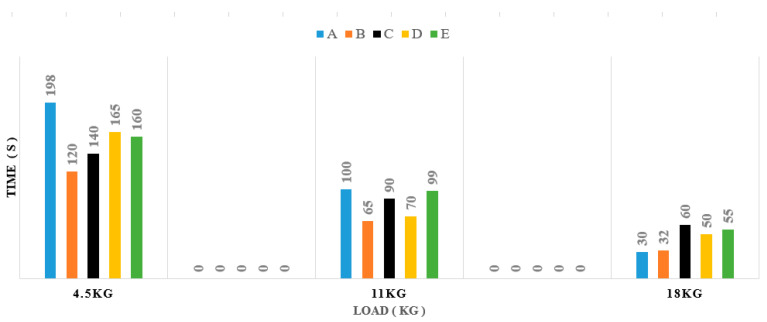
The distribution bar chart time of human fatigue with the load.

After the experiments, we created bar charts for the time and the loads. The time is represented by the parameters T2 which are features of muscle fatigue. The times reduce by the loads, and people increasingly become fatigued with higher loads. Each person’s parameters are different because of their different physical and mental make-ups. T2 is the time the person starts becoming fatigued because the entropy decreases. It means that the spectra of frequency that move into the packet are chosen in wavelet packet transforms. The time linearly changes with the loads during the exercise. Figure 18 is the summary of the time of T2 to present the transition of each person from no-fatigue to fatigue states.

### 3.2. Statistical Analysis

We provide a summary in Table 3 and Table 4 to present details of participation in a variety of loads with duration time of muscle fatigue (T2), normalized EMG entropy, and frequency of machine.

In Table 3, the statistical analysis showed that there was a significant difference of EMG entropy value between the no-fatigue and the fatigue of muscle at difference load because the *p*-value is less than 0.05 (*p*-value at 4.5 kg = 0.009713; *p*-value at 11 kg = 0.001418; *p*-value at 18 kg = 0.004412). For different loads, times of muscle fatigue have significant differences among them, with the *p*-value for load pairs at (4.5 kg, 11 kg) and (11 kg, 18 kg) 0.002182 and 0.009983, respectively. Table 4 is used to confirm the timing of muscle fatigue by the user’s exercise motion frequency (f_m_). This frequency(f_m_) is measured from the IMU sensor, which has significant difference between frequency of muscle fatigue and no muscle fatigue with *p*-value less than 0.05 (*p*-value at 4.5 kg = 0.00885; *p*-value at 11 kg = 0.00689; *p*-value at 18 kg = 0.00668). It concluded that the index_EMG_entropy_ we created can effectively track muscle fatigue at different loads.

To investigate our verification testing and proof of concept, our five young male subjects can be considered sufficient. Further studies with a large group are needed to investigate these findings in a larger group.

## 4. Conclusions

This study provides proof of concept that muscle fatigue can be detected by checking muscle EMG signals, although, so far, our findings are limited to the mere testing of a few young male subjects. Further studies will have to investigate our results in larger user groups. The novelty of our research was detecting muscle fatigue during exercising by checking EMG signals. In this study, we proposed a new index to observe the conditions of people doing exercise. The EMG signal was processed using the wavelet packet entropy to find the EMG entropy that can be applied to detect muscle fatigue. In the proposed method, the packet selected ranges from 62.5 to 125 Hz and is associated with the frequency of muscle fatigue. Combining IMU sensor with EMG sensor to verify the state of a person’s fatigue decreased the power production in response to contractile activity. The new index can be used to detect the conditions of muscle in controlling the smart leg extension machine. Moreover, it also provides insight into the state of the muscle of each person as EMGs differ with people, which depends on the effect of metabolism and environment nutrition. Thus, user conditions during exercise can be thoroughly evaluated to improve the training strength and avoid muscle injury. The EMG signals give information about different behaviors of calf muscle motion. Some actions change mechanical performance capacity, and this results in EMG changes. The effect of fatigue is observable in the recorded EMG signal as a change in the values of selected EMG entropy.

In the future, the new index of muscle fatigue significantly contributes to the exercise process. A person in exercise will know his/her condition of muscle training, so he/she can select the appropriate load, and an intelligent exercise device can also automatically change the load to protect users.

## Figures and Tables

**Table 1:** EMG analysis methods.

Domain	Method
Frequency domain	FFT Autoregressive method Eigenvector
Time-frequency domain	Short-time Fourier transform Wavelet transform Wavelet packet
Time domain	RMS Mean

**Table 2:** Participants.

S/N	Name	Age	Gender
1	A	24	Male
2	B	26	Male
3	C	23	Male
4	D	25	Male
5	E	23	Male

**Table 3:** The properties of muscle fatigue with parameters of time and normalized EMG entropy for each person.

α = 0.05					Standard Deviation of EMG Entropy = 0.000891		
					Standard Deviation Time of Muscle Fatigue = 2		
Subject	EMG Entropy of No-Fatigue	Time of Muscle Fatigue under 4.5 kg (s)	Time of Muscle Fatigue under 11 kg (s)	Time of Muscle Fatigue under 18 kg (s)	EMG Entropy of Muscle Fatigue at 4.5 kg	EMG Entropy of Muscle Fatigue at 11 kg	EMG Entropy of Muscle Fatigue at 18 kg
A	−1	198	100	30	−1.06727	−1.10180	−1.03953
B	−1	120	65	32	−1.06147	−1.06024	−1.06977
C	−1	140	90	60	−1.02911	−1.08497	−1.04079
D	−1	165	70	50	−1.01779	−1.08025	−1.04706
E	−1	160	99	55	−1.04201	−1.12961	−1.09091
*p*-value (no-fatigue–muscle fatigue) at same load					0.009713	0.001418	0.002182

**Table 4:** The motion frequency (f_m_) of non-fatigue and fatigue for each person.

α = 0.05				Standard Deviation Frequency = 0.01 Hz		
Subject	Frequency Non-Fatigue of Machine under 4.5 kg (Hz)	Frequency Non-Fatigue of Machine under 11 kg (Hz)	Frequency Non-Fatigue of Machine under 18 kg (Hz)	Frequency Fatigue of Machine at 4.5 kg (Hz)	Frequency Fatigue of Machine at 11 kg (Hz)	Frequency Fatigue of Machine at 18 kg (Hz)
A	0.35	0.55	0.35	0.25000	0.35000	0.20000
B	0.35	0.55	0.50	0.15000	0.20000	0.15000
C	0.55	0.35	0.35	0.18000	0.20000	0.20000
D	0.56	0.58	0.45	0.24000	0.10000	0.30000
E	0.62	0.60	0.40	0.19000	0.20000	0.20000
*p*-value (no-fatigue–muscle fatigue) at same load				0.00885	0.00689	0.00668
